# Explaining Local Residents’ Attitudes toward Shale Gas Exploitation: The Mediating Roles of Risk and Benefit Perceptions

**DOI:** 10.3390/ijerph17197268

**Published:** 2020-10-05

**Authors:** Liuyang Yao, Qian Zhang, Kin Keung Lai, Xianyu Cao

**Affiliations:** International Business School, Shaanxi Normal University, Xi’an 710119, China; yaoliuyang@snnu.edu.cn (L.Y.); mskklai@outlook.com (K.K.L.); cxy@snnu.edu.cn (X.C.)

**Keywords:** shale gas exploitation, multi-attribute model, generalized structural equation modeling, risk perception, benefit perception, mediation analysis

## Abstract

Using Fishbein’s multi-attribute model, this paper proposes that the impact of socio-demographic and psychosocial factors on local residents’ overall attitude toward shale gas exploitation (SGE) is mediated by their risk and benefit perceptions. The proposition has been validated with the generalized structural equation modeling approach with a cross-sectional dataset of 825 residents from China’s Fuling shale gas field. Results indicate that the influence of benefit perception on residents’ overall attitude outweighs that of risk perception. Moreover, residents’ perceived fairness, affective feeling, and trust in regulatory agencies have positive influences on their overall attitude, primarily via their risk and benefit perceptions, in decreasing order of influences. Finally, we also find that residents’ attitudes have been significantly influenced by their socio-demographic factors, including age, residential area, and political ideology. Thus, our study extends the literature with theoretical and empirical models by exploring the influences factors of local residents’ attitudes toward SGE, and results from our empirical survey provide insight into policy design to promote the acceptance of SGE.

## 1. Introduction

The development of horizontal drilling and hydraulic fracturing has led to the shale boom occurring in the U.S., which significantly boosts natural gas production. This technology has proliferated and is increasingly deployed in countries aside from the U.S., such as Canada, China, and Argentina, each of which has achieved commercial-level shale gas exploitation (SGE). However, conflicting views have emerged regarding the socio-economic and environmental impacts posed by SGE at the local level. On the positive side, some studies found that local residents would benefit from the increments in employment and income caused by SGE [1,2,3,4]. On the negative side, a substantial literature substantiated plenty of localized concerns about SGE’s environmental impacts and health effects [5,6]. Facing these controversies associated with the exploitation activities, the political support of SGE differs widely across nations [7]. At the same time, local residents’ attitudes toward SGE may yet emerge as a critical driver of public policy to ensure that the SGE will be implemented successfully and sustainably [8,9]. Thus, the regulators need to understand the influencing factors involved in local residents’ attitudes toward SGE to help develop more effective action plans that serve the needs and interests of the local community.

The literature has recognized the importance of understanding the determinants of public attitudes toward SGE, which can aid policymakers in making more informed decisions for sustainable SGE [8,10,11]. Survey-based investigations on public attitudes toward SGE, as well as its influencing factors, have attracted considerable research attention. Within the literature, both socio-economic and psychological factors have been linked to the overall attitude, positive attitude (benefit perception), or negative attitude (risk perception) related to SGE [12,13,14,15]. However, despite a mix of qualitative and quantitative approaches employed to explore the factors underlying public attitudes in North American and European countries (e.g., the U.S., the U.K., and Canada), there have been recent calls for more research on this topic in emerging economies, such as China, due to the unique economic and political system in each country [14,15]. Moreover, inspired in previous studies of attitudes toward sustainable technologies [16,17], the overall attitude of SGE should also be determined by the perception of risks and benefits of applying energy technologies. In turn, the perception, as well as the overall attitude, is given causal interpretations by a set of socio-economic (e.g., age and education) and psychological factors (e.g., trust and fairness). With respect to SGE, the complex theoretical relationships among different attitudinal factors (including overall attitude, positive attitude, and negative attitude), socio-economic, and psychological factors remain relatively unexplored, particularly in terms of how they can facilitate the widespread acceptance of, and shape the risk and benefit perceptions about, SGE.

The rapid shale gas development and the considerable concerns with regard to this SGE from local residents call for an improved understanding of local residents’ attitudes toward SGE. The object of this study is to analyze the influencing factors of local residents’ attitudes toward SGE in the context of a rapidly expanding SGE area of China that is characterized by a high population density and lax environmental regulations. Specifically, this study extends the literature with theoretical and empirical models by dividing local residents’ attitudes toward SGE into three dimensions, namely, overall attitude, risk perception and benefit perception. Additionally, we hypothesize that local residents’ risk perception and benefit perception are two mediators in which local residents develop their overall attitude related to SGE, and all dimensions of attitudes are given causal interpretations by a range of literature-based socio-economic and psychological factors. The generalized structural equation modeling, based on a dataset of 825 residents from China’s Fuling shale gas field, has been employed to probe into which influence local residents’ attitudes in this setting.

## 2. Conceptual Framework

### 2.1. Attitudes toward SGE Based on Multi-Attribute Model

Fishbein and Ajzen [18,19] define the attitude toward a given object or concept as a learned predisposition to respond in a consistently favorable or unfavorable manner. Although the literature to date has explored the public’s overall attitude toward SGE [20,21,22], it is more important to understand the formation of the overall attitude considering that an individual’s overall attitude is relatively enduring and difficult to change [16]. Thus, the insights of crucial antecedent factors influencing the overall attitude, such as risk and benefit perceptions [16,17], could be used to develop management strategies to promote residents’ acceptance of SGE. In this regard, the multi-attribute model provides a framework since it explores the multiple aspects of the attitude and explains the overall attitude as a function of beliefs [18,19]. The basic form of the multi-attribute model applied to our study is defined as follows,

(1) $$Attitude=\underset{i}{\sum }\beta_{i}z_{i}$$

where $z_{i}$ is the strength of the residents’ belief in the *i*^th^ impact posed by SGE, and $\beta_{i}$ is the importance weight of the *i*^th^ belief evaluated by the residents. The model thus indicates that the attitude toward a given object or concept is the weighted sum of beliefs ($z_{i}$) about the object and the evaluations ($\beta_{i}$) associated with those beliefs. As suggested by the previous studies [8,21], the Attitude that has been explained in this study is measured by the level of acceptance of SGE. Moreover, similar with previous multi-attribute model applied in other sustainable technologies [16,17], local residents’ beliefs about SGE can be divided into two different types of attitude: risk perception which is a set of negative beliefs linked to SGE’s negative impacts, and benefit perception which is a set of beliefs linked to SGE’s positive impacts.

Thus, based on the multi-attribute model, the overall attitude, i.e., acceptance, is determined by the risk and benefit perceptions about SGE, which are associated with multiple negative and positive beliefs linked to SGE, respectively. Theoretically, local residents’ acceptance of SGE can be improved by their positive beliefs since SGE contributes to local development by increasing employment and income opportunities, improving public infrastructures, and fostering community pride [23,24,25]. Moreover, local residents’ acceptance of SGE can also be weaken by their negative beliefs as SGE poses a level of threat to health and environment, especially when the regulations are not sufficient to address these threatens [25,26,27]. Therefore, the following hypotheses are proposed.

**H1.**  *Local residents’ risk perceptions about SGE negatively influence their acceptance of SGE*.

**H2.**  *Local residents’ benefit perceptions about SGE positively influence their acceptance of SGE*.

### 2.2. Influencing Factors of Attitudes

There has been a rich body of literature that has tested the influences of socio-demographic and psychosocial factors on the attitudes toward SGE [10,11,12,13,14,15,20,21,22,23,24,25,26,27]. Based on a comprehensive framework of energy technology acceptance [16], our research mainly focuses on the following psychosocial factors; (a) residents’ trust in regulatory agencies; (b) their perceived fairness of the SGE; (c) their affective feeling in response to SGE. Moreover, this framework has been extended to incorporate residents’ socio-demographic factors, such as age, education, gender, and income, as suggested by previous shale-related studies. These will therefore be discussed in detail below.

#### 2.2.1. Trust in Regulatory Agencies

When people have insufficient capacities to evaluate and manage risky technology themselves, they form their attitudes based on information provided by other actors who are responsible for the technology. Thus, trust in regulatory agencies is used as a heuristic or alternative ground to evaluate the technology’s risks and benefits and to base their opinions on [16]. For example, in the pro-shale country of China [7], it is intriguing to see that some residents still oppose SGE even after the government has devoted considerable resources to assure the safety of SGE facilities [15]. One of the main reasons might be that these opponents may be less willing to trust regulatory agencies since SGE uses technologies that are relatively new for them. Thus, local residents’ trust may play an essential role in shaping their attitude.

The degree of trust in regulatory agencies has been found to influence the acceptance of a risky technology directly or indirectly via the risk and benefit perceptions [16]. Concerning SGE, empirical studies have found that respondents’ trust is significantly associated with their risk perceptions about SGE [28,29], or their acceptance of SGE [21]. However, the mediational analysis that transmits the effect of trust on acceptance in shale-related research has not received adequate attention. This mediation effect, through risk and benefit perceptions, has already been confirmed in other technologies’ studies, such as nuclear power [30] and agrobiotechnologies [17,31]. As the previous studies imply that a lack of trust in regulatory agencies is related to more risk perception and fewer benefit perceptions about (and thus more acceptance of) the technology, the following hypotheses are proposed.

**H3.**  *Local residents’ trust in regulatory agencies negatively influences their risk perceptions about SGE*.

**H4.**  *Local residents’ trust in regulatory agencies positively influences their benefit perceptions about SGE*.

**H5.**  *Local residents’ trust in regulatory agencies positively influences their acceptance of SGE.*

#### 2.2.2. Perceived Fairness

Fairness can concern various aspects of technology acceptance, of which distributive fairness and procedural fairness are the most thoroughly investigated [16,32]. Distributional fairness focuses on examining to what extent hazardous consequences are equally distributed over people who bear the brunt. Procedural fairness focuses on examining to what extent people are allowed to have access to the decision-making procedure around the hazardous activity. Moreover, the review of Clough [32] on environmental justice and fracking also points out the importance of recognizing the legitimate voice of a full range of stakeholders in SGE’s decision-making process, which is defined as recognition fairness. In the shale-related literature, there has been no empirical analysis to date analyzing the relationship between fairness and acceptance of SGE. In contrast, empirical studies have shown that fairness, as a direct or indirect predictor, is positively associated with the acceptance of other risky technologies [31,33]. Therefore, the following hypotheses are proposed.

**H6.**  *Local residents’ perceived fairness negatively influences their risk perceptions about SGE*.

**H7.**  *Local residents’ perceived fairness positively influences their benefit perceptions about SGE*.

**H8.**  *Local residents’ perceived fairness positively influences their acceptance of SGE*.

#### 2.2.3. Affective Feeling

In addition to trust and fairness, the literature strongly suggests that people rely on a sense of negative or positive gut feelings (i.e., affective feeling) associated with a technology to evaluate its risk and benefit perceptions [34]. A more negative (or positive) feeling toward a new technology may lead people to perceive a higher risk (or benefit) than actually exists as the result of risk (or benefit) seeking. People thus seem to base their attitude toward a new technology on their affective feelings, which has been used a heuristic to evaluate the associated risks and benefits [33]. Although the effect of affective feeling on attitudes toward SGE remains unexplored, results from the studies of other technologies [30,33] support us to make the following hypotheses.

**H9.**  *Local residents’ affective feeling negatively influences their risk perceptions about SGE*.

**H10.**  *Local residents’ affective feeling positively influences their benefit perceptions about SGE*.

**H11.**  *Local residents’ affective feeling positively influences their acceptance of SGE*.

#### 2.2.4. Socio-Demographic Factors

The literature has outlined a number of socio-demographic factors to have influences on local residents’ negative and positive perceptions about SGE and the acceptance of SGE. For example, several studies showed that females tended to perceive higher risks of the SGE [8,20,22,29] and be less supportive of SGE than males [35], and people with conservative political ideology were more likely to support SGE [8,20,22]. However, the impacts of age and education on attitudes vary across different studies. A few studies of SGE found that older people were more likely to perceive fewer risks [28] and be supportive [20,22], while Whitmarsh et al. [8] found that age did not influence the acceptance among the general U.K. population. Last, echoing years of commentary in the studies of information deficit model [36], although the literature of SGE emphasizes the importance of education on public’s attitudes, empirical studies found that education could be a negative predictor of risks perception [14], a positive predictor of acceptance [20], or have no influence on risk perception [28] and acceptance [21,36,37].

The reason for these conflicting results in the literature may be caused by a lack of consistency in the predictors or modeling approaches used to study the demographics’ impacts on attitudes. Thus, this study provides the first attempt to examine the influences of socio-demographic factors together with the psychological factors on the acceptance of SGE, but will not propose any directional hypotheses that suppose the existing relationships.

### 2.3. Mediation Model

**Figure 1:**
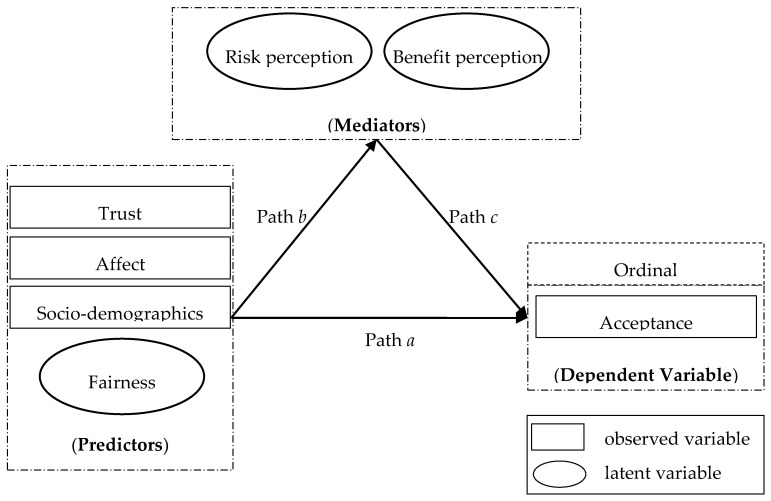
Conceptual model explaining local residents’ attitudes toward SGE.

The preceding discussion concludes a causal flow from trust in regulatory agencies (*Trust*), affective feeling (*Affect*), perceived fairness (*Fairness*), and socio-demographics (*Socio*) to risk perception (*Risk*), benefit perception (*Benefit*), and acceptance (*Acceptance*). These causal relationships, along with relationships between overall attitude and risk and benefit perceptions characterized by the multi-attribute model, provide the conceptual framework for our analysis (Figure 1). In our conceptual framework, the predictors of socio-economic and psychological factors influence *Acceptance* both directly (denoted as Path *a*) and indirectly via risk perception and benefit perception (denoted as Path *b* and Path *c*, separately).

In Figure 1, the overall attitude (*Acceptance*) toward SGE has been measured by one single Likert-type question from “strongly oppose” to “strongly support”, and the responses were numbered ordinally from 1 to 5. Thus, *Acceptance* is an ordered dependent variable in this study. As discussed in Section 2.1, as the risk perception (*Risk*) and benefit perception (*Benefit*) are associated with multiple impacts posed by SGE, *Risk* and *Benefit* have been treated as two latent variables in this study. Similarly, perceived fairness (*Fairness*) is also a latent variable as it contains the dimensions of distributive fairness, procedural fairness, and recognition fairness. 

As Figure 1 indicates, besides a direct causal relationship between the predictors (*Trust*, *Affect*, *Fairness*, and *Socio*) and the dependent variable (*Acceptance*), we propose that the predictors (*Trust*, *Affect*, *Fairness*, and *Socio*) also influence the mediator variables (*Risk* and *Benefit*), which in turn influences the dependent variable (*Acceptance*). Adapted from Baron and Kenny [38], the mediation effects of *Risk* and *Benefit* can be tested with the following regression models.

(2) Model 1: Acceptance=f(Trust, Affect, Fairness, Socio)

(3) Model 2, submodel 21: Acceptance=f(Risk, Benefit, Trust, Affect,Fairness, Socio)

(4) Model 2, submodel 22: Risk=f(Trust, Affect, Fairness,Socio)

(5) Model 2, submodel 23: Benefit=f(Trust, Affect, Fairness, Socio)

Following Baron and Kenny [38], Model 1 will be first estimated to examine whether there is a significant correlation between the predictors and the dependent variable. To include the latent variables (i.e., *Fairness*) as regressors, as well as an ordinal dependent variable (*Acceptance*), the generalized structural equation modeling (GSEM) has been employed. GSEM allow for the simultaneous estimation of the measurement model for the latent variable and the ordered probit model for explaining ordinal dependent variable [39]. Thus, GSEM helps avoid generating biased results from using linear regression models by incorporating the ordered probit model, as well as correct the bias from separately estimating a measurement model and an ordered probit model.

Model 2 is a set of simultaneous equations, including submodel 21, submodel 22, and submodel 23. GSEM has also been employed to simultaneously estimate the measurement model for latent variables (i.e., *Risk, Benefit*, *and Fairness*), estimate the ordered probit model of submodel 21, and estimate the latent variable model of submodel 22 and submodel 23. GSEM has the advantage to provide simultaneous estimations of all three submodels. Results of the GSEM can be used to examine the mediation effects via *Risk* and *Benefit* (i.e., Path b×Path c) through a bootstrap procedure as recommended by Shrout and Bolger [40].

## 3. Survey Design and Data

### 3.1. Survey Procedures

Our survey was conducted in Fuling Shale Gas Field (FSGF), which is China’s first and largest SGD area locating in the country’s southwestern Sichuan Basin. Encouraged by the shale boom in the United States, China started the exploration of shale gas in 2005 to satisfy the nation’s clean energy demand for carbon emission reduction and air quality improvement. It was not until 2014, with the discovery of large-scale FSGF in the Sichuan Basin, that China became the third country to accomplish commercial SGE after the United States and Canada [41]. Since then, China’s annual shale gas production has dramatically increased from 1.3 billion cubic meters (bcm) in 2014 to over 13 bcm in 2019. At the same time, shale gas production in FSGF has dramatically increased from 1.08 bcm to 6.33 bcm, which indicates that FSGF plays a pivotal role in China’s SGE industry. Thus, our survey in FSGF will have a special reference to effective policymaking in promoting the widespread acceptance of SGE.

The survey was implemented through face to face interviews with the households living in four towns, situated in different parts of FSGF: Jiaoshi Town and Baitao Town in areas that were developed from 2013 to 2016, and Jiangdong Town and Pingqiao Town in areas that were developed starting in 2015 and ending in 2020. These four towns varied in population size from 15,000 to 65,000 residents. A simple random sample of 18 villages was selected, and the number of villages in each of the four towns is approximate proportion to the town population size. In each of the 18 selected villages, a simple random sample of 70 households were further selected, which results in a total of 1260 targeted households. We had adopted several measures to ensure the validity of our collected data. First, several pretests were conducted before the full-scale survey in the study areas to ensure that our questionnaire was appropriately designed and modified. Second, our investigators were adequately trained in a two-day course to prepare them for data collection. Last, the invalid responses, including careless responses, rushed-for-time responses and respondents who had difficulty in understanding our questions, were marked as invalid samples by our investigators.

Of the 1260 targeted households, our investigators collected 892 completed answers. Further, there were 85 invalid samples marked by our investigator, and these samples were excluded for further analysis. Thus, the following analysis will include 825 valid questionnaires, 65.48% of the targeted sample.

### 3.2. Measures of the Variables

The measurement scale for *Acceptance*, *Risk*, *Benefit*, *Trust*, *Affect*, and *Fairness* contained 12 specific indicators (Table 1), which were derived from several scales in relevant empirical studies with high reliability and validity [8,21,23,25,26,27,28,29,30,31,32,33]. Moreover, a set of socio-demographic factors, as suggested by previous studies [8,14,20,21,22,28,29,36,37] has also been included for model estimation. Table 1 summarizes the measures and descriptive statistics of these questionnaire items.

As mentioned, as *Risk*, *Benefit*, and *Fairness* include multiple items, they are treated as latent variables. The Cronbach’s alphas of these three latent variables are 0.855 (*Risk*), 0.820 (*Benefit*), and 0.780 (*Fairness*), larger than the acceptable internal consistency of 0.70 [42]. The reliability and validity of each construct are further evaluated with the confirmatory factor analysis (CFA). Results from CFA showed that the composite reliabilities are 0.863 (*Risk*), 0.834 (*Benefit*), and 0.790 (*Fairness*), larger than the recommended level of 0.7 [43], and the average variances extracted are 0.678 (*Risk*), 0.626 (*Benefit*), and 0.557 (*Fairness*), larger than the recommended level of 0.5 [43]. Thus, the latent variables measured in this study had good reliability and validity.

### 3.3. Sample Characteristics

As Table 1 shows, most of our respondents were male because household heads were the first-preference interviewee to be representative of their household. The average age and education level of our sample were able to portray the demographic characteristics in SGE areas of southwest China [14,22], largely locating in China’s rural areas. Moreover, 57.1% of our respondents were from the areas of early exploration stage, which was consistent with the population proportion (56.8%) of this area. Having household members belonging to the Chinese Communist Party (CCP) was used as a proxy for political ideology, and results indicate that about 1/4 of the respondents’ families had CCP members. The average personal annual income ranged from 9000 to 12,000 yuan, a little lower than the official statistics (14691 yuan), which was probably due to the common underreported income problem during the field survey [44]. 

Table 1 also shows a high level of acceptance of local SGE in FSGF, which had no significant differences with the study of Yu et al. [21] conducted in another part of southwest China. The risk perception is reflected by respondents’ moderate concerns about the health risks, above moderate concerns about the environmental risks, and above moderate concerns about the inadequate regulatory enforcement. The benefit perception was reflected by respondents’ recognition of the infrastructure improvements, basically agreeing with the increasing job and income opportunities, and undecided agreement on the enhancement of community pride. The means of residents’ trust in regulatory agencies and affective feelings were around 3.5, larger than all dimensions of their previewed fairness. The means of different dimensions of fairness indicate that participants perceived relatively low levels of recognition fairness, distributive fairness, and procedural fairness in decreasing order. The reason might be that China’s Environmental Impact Assessment Law gave the public a legitimate seat in participating in the formulation of policies and regulations, while the procedure was not transparent [45]. 

## 4. Estimation and Results

Table 2 presents the results of GSEM to handle the ordinal dependent variable and latent variables. The goodness-of-fit of each model (in the lower part of Table 2) shows that GSEM fits the data well compared with the null parameter model. Moreover, estimates from the measurement models of GSEM (in the middle part of Table 2) show that all factor loadings are around the value of 1, which indicates a good construct validity for each construct [43], consistent with results of CFA in Section 3.2.

Without accounting for risk and benefit perceptions, Model 1 shows the role of exogenous predictors in shaping residents’ acceptance of SGE. All three psychological variables (*Trust*, *Fairness*, and *Affect*) have significant and positive influences on *Acceptance*. For the socio-demographic factors, age (0.006 with *p* = 0.047) is positively related to *Acceptance*, and households having CCP members (0.150 with *p* = 0.047) or living in areas of the early exploration stage (0.146 with *p* = 0.064) are more supportive of SGE. In contrast, other socio-demographic factors have no significant influences on *Acceptance*.

Model 2, compared with Model 1, has been estimated to examine whether risk and benefit perceptions mediate the effects of the predictors on *Acceptance*. First, there are significant opposite influences from the mediators on *Acceptance* (submodel 1), which confirms our hypotheses H1 and H2. Second, comparing the results from submodel 1 and Model 2, the impacts of all three psychological factors decrease noticeably after incorporating the mediation effects. Third, the indirect paths (submodel 22 and submodel 23) from all three psychological factors to *Acceptance* are significant. The joint significance of each indirect path has been tested with a 1000 times bootstrap procedure. Bootstrap results show that *Acceptance* has been significantly and indirectly influenced by *Trust* (0.102 with *p* = 0.000), *Fairness* (0.288 with *p* = 0.000), and *Affect* (0.110 with *p* = 0.000). Thus, our results confirm that the impacts of *Trust*, *Fairness*, and *Affect* on *Acceptance* are mediated by residents’ risk and benefit perceptions. For the socio-demographic factors, participates who are female are more likely to feel fewer risks posed by SGE, and results from the bootstrap indicate that being a female has a negative indirect effect on *Acceptance* (0.048 with *p* = 0.000). However, the other indirect paths from the socio-demographic factors to the mediators are not significant. 

Results from Model 2 also confirm that *Trust*, *Fairness*, and *Affect* are positively related to *Acceptance*, negatively related to risk perception, and positively related to benefit perception. As a result, our hypotheses from H3 to H11 are supported. Moreover, after incorporating the mediating effects of risk and benefit perceptions, the direct effects of *Affect, age,* and *location* on *Acceptance* are still significant, while the direct effects of *Trust* and *Fairness* on *Acceptance* are no longer significant. In other words, *Affect* have both direct and indirect influences on *Acceptance* (with the total effect of 0.188 with *p* = 0.000); *age*, *communist*, and *location* only have direct influences on *Acceptance*; and *Trust, Fairness* and *gender* only have indirect influences on *Acceptance*. 

## 5. Discussion

The results of GSEM have confirmed the mediating roles of risk and benefit perceptions on explaining local residents’ attitudes toward SGE. The main findings are discussed as follows.

Our results indicate that models with or without considering the mediation effects generate similar results of the relationship between the predictors and independent variables, which demonstrate the robustness of the results derived from GSEM. As hypothesized, perceived risk (−0.232) negatively influences the acceptance toward SGE, and perceived benefit (0.538) positively influences the acceptance toward SGE. Thus, local residents will be more supportive if they feel fewer risks and more benefits from the nearby SGE. Moreover, the impact of risk perception on acceptance is significantly weaker than that of benefit perception. In other words, local residents’ overall attitude toward SGE based mainly on the benefits delivered by SGE rather than the associated risks. Similar findings can also be seen in the study of Yu et al. [21] using a regression model. The reason might be that residents living in less developed regions, like FSGF, tend to pay more attention to the benefit rather than the risks about the potentially hazardous facility in their communities [46,47]. Another possible reason is that most of the information received by local residents has focused more on the positive rather than the negative impacts of SGE [7], which also makes the residents pay more attention to the positive aspects of SGE.

All three psychological predictors have positive influences on attitude. This is a common finding in the literature, as discussed in Section 2.2, suggesting that residents who have higher levels of trust in regulatory agencies, perceived fairness, and positive feeling related to SGE, tend to have more acceptance of SGE. Our results also imply that, after controlling the mediation effects, *Trust* and *Fairness* have no direct influences on the overall attitude, while *Affect* has both direct and indirect influences. Thus, results from this study contribute to the debate on exploring the ways in which the psychological factors are (directly or indirectly) related to attitudes [16,30]. Moreover, among all three psychological predictors, *Fairness* has the largest influence on the overall attitudes, followed by *Affect* and *Trust*. This result highlights the importance of ethical dimensions for promoting the acceptance of local SGE, as suggested by Clough [32].

For the socio-demographical factors, our results show that *age* is positively linked to the overall attitude, which is consistent with previous studies [20,22]. The impact of *location* is inconsistent with the finding of Boudet et al. [20], who confirmed that people seem to be more supportive of SGE in areas that have ongoing projects existed for a long time. The effect of *communist* is inconsistent with the finding of Yu et al. [21] as CCP members are more likely to support the nation’s will to promote SGE by top-down governance. The influence of gender in Model 1 is not significant. However, in Model 2, gender is negatively linked to risk perception, which leads to more supportive of SGE. This result is different from previous studies who find females tended to perceive lower risks about and more supportive of SGE [8,20,22,29]. One possible explanation is that females are more easily to be affected by the propaganda campaign [47], and China’s pro-shale development propaganda [7] has alleviated their concerns about SGE.

## 6. Conclusions

Based on the multi-attribute model, this study has examined whether risk and benefit perceptions mediate the effects of socio-demographic and psychosocial factors on attitudes toward SGE. The proposed hypotheses have been tested based on a cross-sectional dataset of 825 residents from FSGF, southwest China. To deal with an ordered dependent variable and influences from latent variables, generalized structural equation modeling (GSEM) has been used to estimate the mediation effects of local residents’ risk and benefit perceptions on the influences of their acceptance of SGE from trust, perceived fairness, affective feelings, and a set of socio-demographic variables.

All the proposed hypotheses have been supported. Our results indicate that local residents’ acceptance of SGE depends on their perceptions of the risky and beneficial attributes associated with the exploitation. Most importantly, the influence of benefit perception on acceptance outweighs that of risk perception, which has several policy implications for promoting the acceptance of SGE. For example, regulatory agencies and shale companies should pay more attention to providing easily perceived benefits, and such approaches include enhancing community image, increasing the positive side of media’s SGE coverage, and improving local infrastructure. Additionally, approaches that help to emphasize the safety of the SGE facilities should also be considered, such as enhancing the legislation and regulation for SGE, as well as the health risk communications between experts and local residents.

Furthermore, our results suggest that perceived fairness, affective feeling, and trust have positive influences on the overall attitude, in decreasing order of influence. Thus, meaningful policy implications can be deduced to enhance the residents’ previewed fairness, affective feeling, and trust associated with SGE. For example, local government should promote residents’ perceived fairness with regard to attending local residents’ legitimate voices, disseminating real-time information, establishing additional detailed procedures for selecting well locations during SGE. In an effort to address residents’ affective feelings, governments and shale companies can increase residents’ positive impressions through various initiatives, such as investing in more environmentally friendly technologies, increasing pro-shale development propaganda, and appropriately responding to negative publicity or rumors. To increase the residents’ trust in regulatory agencies, local authorities could take measures to demonstrate the benefits of SGE, partner with local communities, and listen to local needs.

Finally, our research on the attitudes toward SGE provides several further directions. First, as environmental and health concerns grow with China’s expanding SGE activities and their associated infrastructure, further exploration can explore the attitudes of the general public [8], who are indirectly affected by SGE and may have different views with the local residents. Second, local residents’ attitude toward SGE may differ across countries because of the difference in political support of SGE in different countries [7]. Thus, the research framework proposed in this study should be tested further with a greater number of individuals to explore the cross-cultural validity of our research conclusions. Third, as SGE poses multiple threats and benefits to the surrounding population [5,48], further research should identify and prioritize different SGE’s impacts on local communities to better improve residents’ risk and benefit perceptions.

## Figures and Tables

**Table 1:** Definitions and descriptive statistics of variables for model estimation.

Factor	Description	Mean	S.D.
*Acceptance ^a^*	Accept shale gas exploitation (SGE) in local community.	4.046	1.019
*Risk ^b^*	Be troubled by the negative environmental impacts (*i1_r*).	3.444	1.282
	Be concerning about the potential hazard to human health (*i2_r*).	2.992	1.400
	Current regulations are not sufficient to prevent SGE’s risks (*i3_r*).	3.679	1.079
*Benefit ^b^*	SGE provides job and income opportunities for residents (*i1_b*).	3.724	0.976
	SGE facilitates local infrastructure construction (*i2_b*).	4.006	0.993
	Residents’ sense of community pride has been enhanced (*i3_b*).	3.193	1.351
*Trust ^b^*	Trust the information given by scientists and authorities.	3.439	1.225
*Fairness ^b^*	Residents who bear the risks of SGE are properly compensated (*i1_f*).	2.143	1.143
	SGE has transparent procedures in risk control (*i2_f*).	1.842	0.994
	There are effective ways to raise concerns about SGE (*i3_f).*	3.170	1.373
*Affect ^c^*	Have a negative to the positive feeling of SGE in local community.	3.554	1.342
*Age*	Age of the surveyed respondent.	47.272	14.900
*Gender*	Gender of the surveyed respondent: Female = 1; Male = 0.	0.112	0.315
*Income ^d^*	Average personal annual income: less than 3K = 1; (3K, 6K) = 2; (6K, 9K) = 3; (9K, 12K) = 4; (12K, 15K) = 5; (15K, 18K) = 6; above 18K = 7.	3.686	1.769
*Education*	Education level of the respondent: Primary school or under = 1; Junior high school = 2; Senior high school =3; College degree or above = 4.	1.722	0.838
*Communist*	Having Chinese Communist Party members: Yes = 1; No = 0.	0.262	0.440
*Location*	Locating in the early exploration stage of the area: Yes = 1; No = 0.	0.571	0.495

Note: (a) Measured by “strongly oppose = 1”, “oppose = 2”, “neutral = 3”, “support = 4”, and “strongly support = 5”; (b) Measured by “not at all = 1”, “small extent = 2”, “moderate extent = 3”, “great extent = 4”, and “strongly agree = 5”; (c) Measured by a 5-point Likert-type scale including “strongly negative = 1”, “negative = 2”, “neither negative nor positive = 3”, “positive = 4”, and “strongly positive = 5”; (d) measured by thousand Yuan (1000 Yuan = 151 USD), denoted as K.

**Table 2:** Results of the generalized structural equation modeling.

	Variables	Model 1		Model 2					
		Coef.	*p* > |z|	Coef.	*p* > |z|	Coef.	*p* > |z|	Coef.	*p* > |z|
***Structural***		*Acceptance*		submodel 21 *Acceptance*		submodel 22 *Risk*		submodel 23 *Benefit*	
	*Risk*			**−0.227**	0.000				
	*Benefit*			**0.540**	0.000				
	*Trust*	**0.131**	0.000	0.040	0.249	**−0.191**	0.000	**0.109**	0.000
	*Fairness*	**0.306**	0.000	0.040	0.561	**−0.455**	0.000	**0.342**	0.000
	*Affect*	**0.177**	0.000	**0.080**	0.013	**−0.176**	0.000	**0.129**	0.000
	*age*	**0.006**	0.047	0.006	0.063	0.001	0.771	0.001	0.576
	*communist*	0.150	0.095	0.174	0.059	0.099	0.210	0.008	0.893
	*gender*	0.088	0.482	0.032	0.803	−0.213	0.053	0.023	0.797
	*education*	0.070	0.176	0.031	0.558	−0.044	0.335	0.054	0.133
	*location*	0.146	0.064	**0.200**	0.014	0.088	0.211	−0.045	0.415
	*income*	−0.012	0.579	−0.014	0.548	0.001	0.942	0.001	0.956
***Measure***		*Fairness*		*Risk*		*Benefit*		*Fairness*	
	*i1*	1		1		1		1	
	*i2*	0.909	0.000	1.074	0.000	0.957	0.000	0.920	0.000
	*i3*	1.270	0.000	0.887	0.000	1.346	0.000	1.283	0.000
***Fitness***	LLF	−4525.75		−11022.94					
	AIC	9094.33		22165.88					
	BIC	9198.07		22448.80					

Note: (a) Bold and underlined coefficients are significant at 1%, bold coefficients are significant at 5%, and underlined coefficients are significant at 10%. (b) *i1*, *i2*, and *i3* are different indicators for the corresponding latent variables, as defined in the description column of Table 1. (c) Fitness of the model is evaluated by the log-likelihood function (LLF), Akaike information criterion (AIC), and Bayesian information criterion (BIC).
